# Roles of Polyploid/Multinucleated Giant Cancer Cells in Metastasis and Disease Relapse Following Anticancer Treatment

**DOI:** 10.3390/cancers10040118

**Published:** 2018-04-15

**Authors:** Razmik Mirzayans, Bonnie Andrais, David Murray

**Affiliations:** Department of Oncology, University of Alberta, Cross Cancer Institute, Edmonton, AB T6G 1Z2, Canada; bonnie.andrais@ahs.ca (B.A.); david.murray5@ahs.ca (D.M.)

**Keywords:** polyploid/multinucleated giant cells, metastasis, anticancer treatment, cancer relapse

## Abstract

Tumors and tumor-derived cell lines contain polyploid giant cells with significantly elevated genomic content, often with multiple nuclei. The frequency of giant cells can increase markedly following anticancer treatment. Although giant cells enter a dormant phase and therefore do not form macroscopic colonies (aggregates of ≥50 cells) in the conventional in vitro colony formation assay, they remain viable and metabolically active. The purpose of this commentary is to underscore the potential importance of polyploid/multinucleated giant cells in metastasis and cancer recurrence following exposure to anticancer agents. We also discuss the possibility that most preclinical (cell-based and animal model) drug discovery approaches might not account for delayed responses that are associated with dormant giant cells.

## 1. Introduction

**Figure 1:**
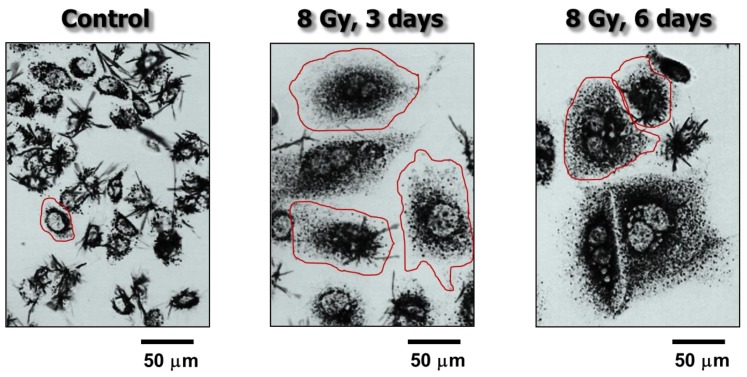
Bright-field microscopy images showing the metabolic activity of MCF7 (p53 wild-type) breast carcinoma cells before (control) and at 3 or 6 days after exposure to ionizing radiation (8 Gy). Metabolic activity was measured by the ability of the cells to convert the yellow 3-(4,5-dimethylthiazol-2-yl)-2,5-diphenyl-tetrazolium bromide (MTT) agent to its formazan metabolite (dark granules and crystals). Images were acquired after incubation of cells with MTT for ~1 h. The border of some cells is marked for clarity. Data taken from Mirzayans et al. [12].

The involvement of the p53 tumor suppressor protein in the DNA damage surveillance network was established in the early 1990’s. This discovery led to a model in which p53 signaling either promotes survival by activating cell cycle checkpoints to facilitate repair or induce apoptotic cell death (e.g., [1,2,3]). This model is still being widely cited and the concepts embodied therein have been important drivers of research in the field of DNA damage response. However, as discussed recently [4,5], numerous studies reported in the past two decades have established the presence of a threshold mechanism for stress-induced apoptosis in most human cell types, independent of their p53 status. Importantly, a large body of evidence from studies with solid tumors and solid tumor-derived cell lines demonstrated that a major response triggered by moderate, clinically relevant doses of cancer therapeutic agents is a sustained proliferation arrest (dormancy), rather than apoptosis [6,7,8,9,10,11,12,13,14,15,16,17,18]. A significant proportion of dormant cells remain viable and metabolically active for long times (weeks) post-treatment [12,13,14] (also see Figure 1).

**Figure 2:**
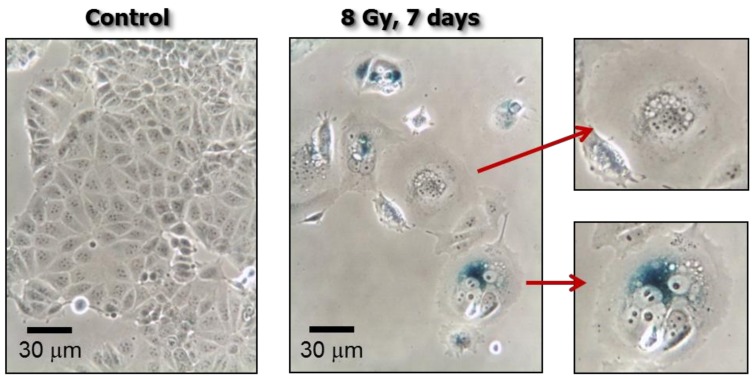
Phase-contrast photomicrographs showing stress-induced premature senescence (SIPS) in MDD2, a mutant p53-expressing derivative of the MCF7 breast carcinoma cell line [19]. Cultures were exposed to ionizing radiation (8 Gy) or sham-irradiated (control), incubated for seven days, and evaluated for flattened and enlarged cellular morphology and positive (blue) staining in the senescence-associated *β*-galactosidase assay.

This proliferation arrest is often accompanied by markedly increased cell size, which can reflect stress-induced premature senescence (SIPS) and/or the development of polyploid “giant” cells with either a highly enlarged nucleus or multiple nuclei. Cells undergoing SIPS are typically identified by senescence-associated β-galactosidase (SA-β-gal) staining. As shown in Figure 2, SA-β-gal positive and negative multinucleated giant cancer cells can arise in the same culture. Irrespective of their SA-β-gal staining status, proliferation arrested (dormant) cancer cells secrete tumor promoting factors and they can give rise to progeny with stem cell-like properties [4,5]. 

The purpose of this commentary is to underscore the potentially significant roles that are played by polyploid/multinucleated giant cancer cells in metastasis and cancer relapse following anticancer treatment, and to discuss the possibility that giant cancer cells and their tumor repopulating descendants might not be accounted for in assays that are widely used for preclinical assessment of agents with anticancer properties. 

## 2. Tumor Cell Heterogeneity

Tumors are complex systems that include heterogeneous cancer cells with markedly differing sizes and genomic contents (reviewed in [20]). These encompass bulk cells, stem cells, and polyploid cells. The bulk of cancer cells within the majority (~90%) of solid tumors are aneuploid; such cells have an alteration (often gain) of chromosome number that is not a multiple of the diploid (2n) component. Cancer stem cells are often much smaller than the bulk of cells, whereas polyploid cells are larger than bulk cells by virtue of their increased ploidy (>4n). 

Coward and Harding [21] developed a rigorous flow cytometry approach for the size distribution measurement of a large number of cells (e.g., 100,000) under conditions that eliminate the confounding effects of subpopulation size and cell number variation between different samples. For low-passage primary cell lines that were established from the tumors of 10 glioblastoma patients, the lowest frequency of polyploid cells was 1 in 20 cells (i.e., 5% of total cells). Based on this observation, the authors estimated that brain tumors with volumes of ~1 cm^3^ will contain at least five million polyploid cells. This flow cytometry approach does not distinguish between mononucleated (polyploid) and multinucleated giant cells.

**Figure 3:**
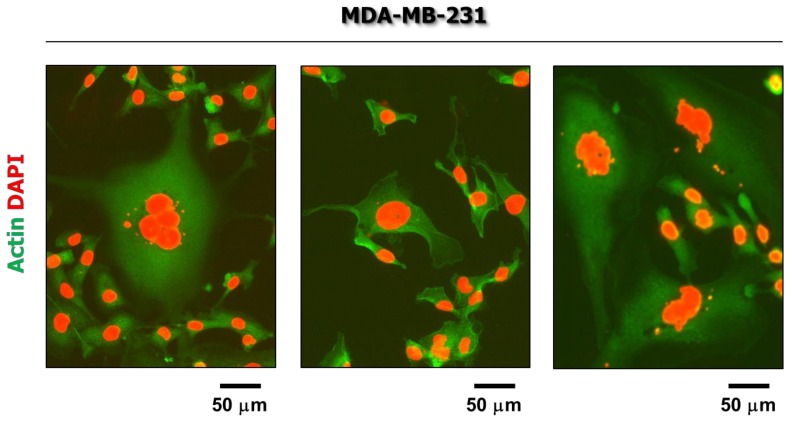
Fluorescence images showing the presence of giant cells in non-stressed cultures of the MDA-MB-231 breast carcinoma cell line.

Not surprisingly, cultures of established solid tumor-derived cell lines are also heterogeneous. Employing immunofluorescence microscopy, we have observed that spontaneously arising giant cells with significantly elevated genomic content are particularly enriched in some breast carcinoma cell lines that express mutant p53 (e.g., MDA-MB-231; Figure 3).

**Figure 4:**
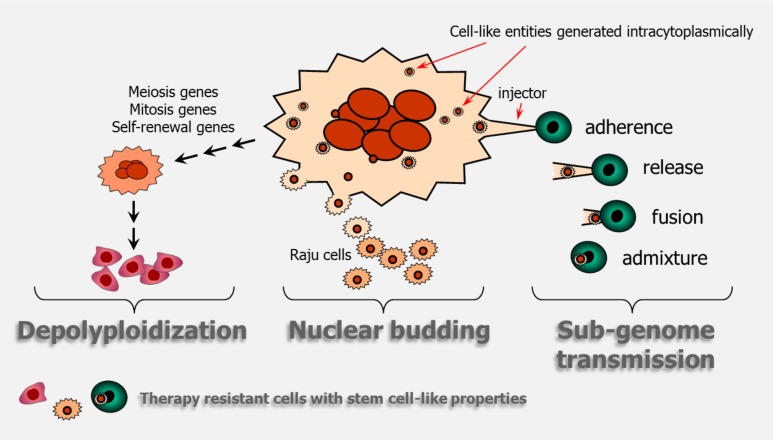
Cartoon illustrating giant cancer cell-mediated stemness through depolyploidization, nuclear budding, and sub-genome transmission. The cartoon on sub-genome transmission is adapted from Figure 7A of Díaz-Carballo et al. [41].

Giant cells with an elevated nuclear content either cease to proliferate or proliferate very slowly such that they are often scored as “dead” in the conventional colony formation assay. However, it has been known for decades that at least a subset of giant cells is anything but dead! In addition to secreting growth factors, giant cells can promote stemness through: (i) nuclear budding or bursting, similar to simple organisms like fungi; (ii) depolyploidization; and, (iii) horizontal transmission of a “sub-genome” between cells (also see Figure 4). 

The discovery that multinucleated giant cells in higher eukaryotes are capable of continuously generating rapidly proliferating mononucleated cells by a budding process was first reported for avian hematopoietic macrophage cultures by Solari et al. [22] in 1995; the link between nuclear budding and transformation was demonstrated for cultured marsupial, snail and human cells by Walen [23] in 2002, and for cultured murine and human cells by Sundaram et al. [24] in 2004. This bizarre mode of cell division was called “neosis”, and the resultant tumor-initiating cells were called “Raju” [24]. This parasexual mode of somatic reduction division of giant cells has since been reported for different mammalian cell types [25,26,27,28,29,30], including human ovarian [31,32], breast [33], colon [34], and prostate [35] cancer cell lines. According to Niu et al. [36], giant cancer cells exhibit self-renewal via endoreduplication and further undergo nuclear budding or bursting to give rise to small daughter nuclei; these nuclei then acquire cytoplasm, split off from the giant mother cells, and exhibit long-term proliferation. The authors referred to this process as the “giant cell cycle.”

Depolyploidization of polyploid/multinucleated giant cells was first reported by Erenpreisa et al. [37] and Illidge et al. [38] in 2000. These and subsequent studies demonstrated that giant cells first undergo a ploidy cycle, which is regulated by key mediators of meiosis (e.g., MOS), mitosis (e.g., aurora B kinase), and self-renewal (e.g., OCT4), ultimately resulting in the emergence of a para-diploid progeny, containing a near-diploid number of chromosomes, which exhibit mitotic propagation (reviewed in [39,40]).

In addition, very recent work of Díaz-Carballo et al. [41] has documented that multinucleated cancer cells can promote stemness of *surrounding* cells via a sub-genome transmission. In this process, giant cells intracytoplasmically generate daughter cells that express high levels of cancer stem cell markers which are then transferred into surrounding cells via cytoplasmic tunnels, conferring the recipient cells with stem cell properties. 

## 3. Roles of Giant Cancer Cells in Metastasis and Therapy Resistance

Zhang et al. [42] reported studies with the PC-3 human prostate cancer cell line, demonstrating that polyploid/multinucleated giant cells are more aggressive and metastatic than parental cells. The authors injected green fluorescence protein (GFP)-expressing PC-3 cells in the footpad of nude mice, which resulted in metastasis to inguinal lymph nodes. The metastasized PC-3 cells were collected from the lymph nodes and were reinjected in the footpads of healthy nude mice. This process was repeated for six cycles, after which the metastasized cells were collected. These cells were called PC-3-GFP-LN. Polyploid/multinucleated giant cells were enriched with each selection cycle and became predominant in the PC-3-GFP-LN cell line. The majority of giant cells were multinucleated, with some containing as many as 22 nuclei per cell. The PC-3-GFP-LN cell line potently developed metastasis in the lung, bone, inguinal node, and cervical node. Furthermore, the PC-3-GFP-LN cell line was highly resistant to the chemotherapeutic drugs cisplatin, doxorubicin, and 5-fluorouracil when compared to the parental PC-3 cell line [42]. (Another important property of the PC-3-GFP-LN cell line will be considered in Section 6 below.)

Weihua et al. [43] used in vitro and in vivo approaches to characterize multinucleated cells that arise spontaneously in the murine fibrosarcoma cell line UV-2257. Employing live cell imaging, the authors showed that: (i) a single mononuclear cell could undergo multinucleation because of the absence of cytokinesis; and, (ii) a single multinucleated giant cell could produce four multinucleated giant cells in one round of cell division. Giant cells were more resistant to doxorubicin than mononuclear cells. Furthermore, giant cells exhibited the ability of self-renewal and formed colonies when seeded in hard agar, indicating anchorage independent proliferation. After a sequential passage of UV-2257 cultures through nylon meshes of different sizes, these authors were able to separate multinucleated giant cells from bulk cells. This approach enabled them to determine the tumorigenic potential of individual giant cells when being grafted under the skin of athymic nude mice (NCI-nu). Grafting only a single giant cell was sufficient to produce orthotopic and metastatic (lung) tumors in this murine fibrosarcoma model [43].

The number of reports demonstrating the relationship between polyploidy and cancer is increasing. Hasegawa et al. [44], for example, reported studies with mouse models demonstrating that multinucleated giant cancer cells and cancer-associated fibroblasts were related to peritoneal metastasis of pancreatic cancer. Furthermore, several studies involving different cancer cell types have demonstrated that polyploidy facilitates epithelial to mesenchymal transition (EMT) [21,32,33,40,45,46]; EMT is a complex molecular and cellular process that plays a key role in cancer metastasis and progression, as well as resistance to a variety of therapeutic agents (reviewed in [21]). Shu et al. [47] have recently discussed the dark sides of polyploidy in the context of primary tumor formation, cancer progression, and metastasis. 

## 4. Roles of Giant Cancer Cells in Disease Relapse after Anticancer Treatment

The proportion of polyploid/multinucleated giant cancer cells both in vitro and in vivo increases markedly under stressful conditions. This increase can be triggered by replicative stress [48] and hypoxia [31,32,33,34], which occur in the tumor microenvironment in the absence of exogenous stress, as well as after exposure to ionizing radiation [6,12,13] and chemotherapeutic drugs, such as cisplatin [7,14], doxorubicin [9,10,11], paclitaxel [36,46], docetaxel [49], 5-fluorouracil, and irinotecan [11]. Below, we will consider representative reports demonstrating that the creation of viable and metabolically active giant cells following genotoxic stress is not an infrequent response in solid tumors and solid tumor-derived cell lines, and that the progeny of giant cells may contribute to cancer recurrence following anticancer treatment.

### 4.1. Enrichment of Viable and Metabolically Active Giant Cancer Cells Following Exposure to Anticancer Agents

**Figure 5:**
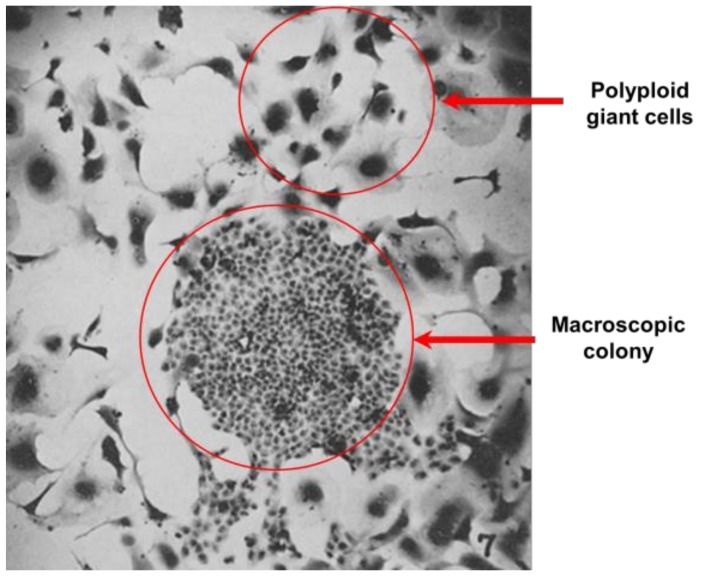
A phase contrast microscopy image showing remarkable (~10 times) size differences between proliferating (colony forming) cells and proliferation arrested polyploid giant cells. The image was taken, with permission, from the original work of Puck and Marcus that was published in 1956 [6], reporting the effect of ionizing radiation (9 Gy) on the colony forming ability of HeLa cell cultures.

The observation that genotoxic stress can trigger the development of giant cells was first reported by Puck and Marcus for the human HeLa cervical carcinoma cell line that was exposed to ionizing radiation. In their seminal paper that was published in 1956 [6], the authors not only established the experimental conditions for the colony formation assay, which has since become the gold standard for evaluating radiosensitivity/chemosensitivity in cultured mammalian cells, but also reported detailed evaluation of cells that do not form macroscopic colonies of at least 50 cells within ~10 days after irradiation. The following observations regarding cells that were “killed” by radiation should be noted:

- Cells that had lost the ability to reproduce (i.e., form a macroscopic colony) following exposure to radiation doses below 8 Gy could still multiply several times, whereas in response to higher doses even one cell division was precluded. (Under their experimental conditions, exposure to a 7-Gy dose of radiation resulted in loss of colony forming ability in >95% of cells.)
- A large proportion of cells that did not form a colony after exposure to any dose of radiation gave rise to one or more giant cells with extremely enlarged morphology (also see Figure 5).
- These giant cells metabolized at a high rate, as judged by their ability to change the pH of the growth medium; they could be maintained in the metabolically active state for long periods (e.g., three weeks) if the medium was regularly replenished.
- Some of the irradiated cells disappeared from the culture dish, presumably due to disintegration. Importantly, the authors noted that “this action of radiation is by far the least efficient, since even after 10,000 r (100 Gy), 5–10% of the original cell inoculum is recoverable as giants.”

**Figure 6:**
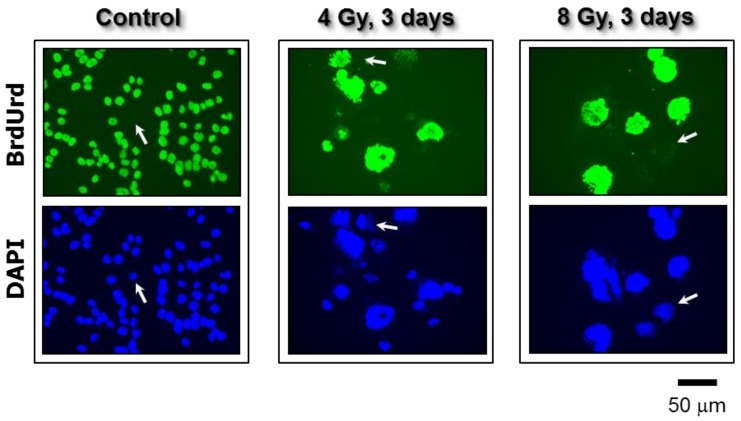
Fluorescence images showing proliferating cells (BrdUrd-positive; green) and total cells (DAPI; blue) in cultures of the p53 knock out HCT116 colon carcinoma cell line. Images were acquired before and three days after exposure to ionizing radiation. In all of the experiments, BrdUrd was added to the culture medium for the final 24 h of the incubation period to allow for its incorporation into genomic DNA. Arrows show some cells that did not incorporate BrdUrd under these conditions. All images were acquired at the same magnification. Taken from Mirzayans et al. [12].

We have recently reported similar observations with other widely-used cancer cell lines after exposure to moderate (“clonogenic survival-curve-range”) doses of ionizing radiation (e.g., 8 Gy) [12,13] or the chemotherapeutic drug cisplatin (e.g., 10 μM) [14]. We demonstrated that exposure of cancer cell lines lacking wild-type p53 function to these agents followed by incubation for three days resulted in polyploidy/multinucleation in a large proportion (>50%) of cells. Single-cell observations revealed that virtually all cells—irrespective of their morphology and size—that remained adherent to the culture dish for the duration of the experiments (up to three weeks post-treatment) retained membrane integrity and exhibited the ability to metabolize the tetrazolium salt MTT to its water-insoluble formazan derivative (also see Figure 1). The majority (>60%) of polyploid/multinucleated giant cells exhibited ongoing DNA synthesis, as judged by bromodeoxyuridine (BrdUrd) incorporation in the nucleus (also see Figure 6).

Was et al. [11] reported the long term (>3 weeks) chemotherapeutic responses of the human colon carcinoma cell lines HCT116 and SW480, evaluated in vitro for different end points, including proliferation arrest, morphology, stemness, and the resumption of proliferation. Cultures were treated with a given drug (5-fluorouracil, irinotecan, or doxorubicin) for different cycles to mimic the therapeutic regimes used clinically. Each cycle involved 24 h treatment with a drug, followed by three days of incubation in fresh medium without drug. This pulse-chase cycle was repeated for six times (LONG CHEMO protocol); alternatively, after three cycles cells were cultured in drug-free medium for two weeks (AFTER CHEMO protocol). Under these conditions, the treatment of HCT116 and SW480 cultures with any of the three chemotherapeutic drugs resulted in SIPS, as evident from sustained proliferation arrest, markedly increased cell size, polyploidization, augmented SA-β-gal activity, and the senescence-associated secretory phenotype. A subset of cells undergoing SIPS exhibited features of stemness, including the elevated expression of NANOG and CD24. Furthermore, the proliferation-arrested response in drug-treated cultures was followed by resumption of proliferation which was largely attributed to the progeny of polyploid giant cells. These and related observations led the authors to conclude that “certain clinically used drugs induce senescence in colon cancer cells. Some senescent cells may display a specific phenotype being a combination of stem-like and differentiated cell features, which makes them tumor-initiating cells. Therefore, we propose that senescence of cancer cells should be carefully considered as a therapy-resistance mechanism …”

### 4.2. Contribution of Giant Cancer Cells to Tumor Regrowth in Response to Cytotoxic Therapy

**Figure 7:**
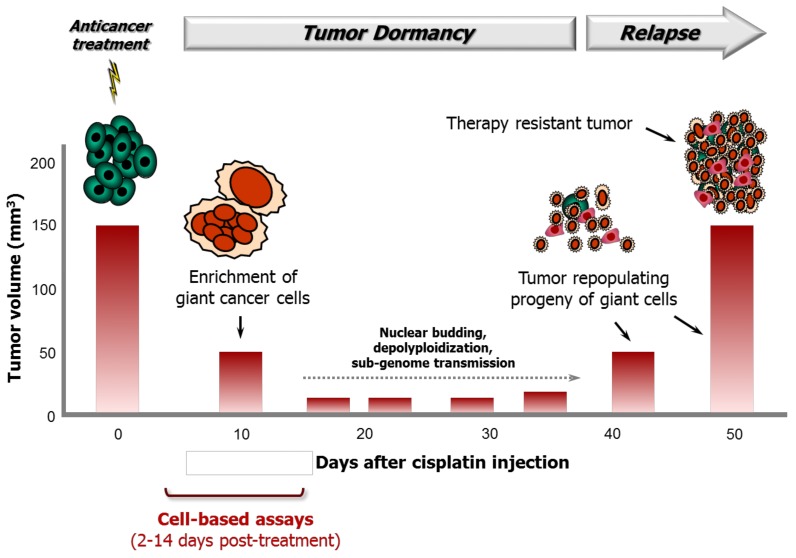
Cartoon illustrating the role of giant cancer cells in tumor response to cisplatin treatment in a syngeneic rat colon carcinoma model reported by Puig et al. [7]. Although cisplatin treatment results in initial tumor shrinkage and a dormant state up to ~35 days post-treatment, it also triggers the creation of polyploid/multinucleated giant cells that give rise to therapy resistant progeny that repopulate the tumor.

Puig et al. [7] used inbred BD-IX rats that were subcutaneously injected with syngeneic PROb colon carcinoma cells to characterize the role of polyploid giant cancer cells in therapy response following cisplatin treatment. The study was performed using rats that were bearing tumors with a mean volume of 132 mm^3^ and cisplatin concentrations that did not result in toxic side effects. The authors made several intriguing observations of potential clinical relevance, some of which are noted below. Tumor volumes reported for control and cisplatin-treated rats are shown in Figure 7.

- Intraperitoneal injection of animals with cisplatin at a maximum tolerated dose resulted in significantly delayed tumor progression but did not lead to tumor elimination.
- The tumors gradually shrank to <50 mm^3^ and remained in a dormant state between ~10 and ~40 days after cisplatin treatment.
- At the histological level, at 10 days after cisplatin treatment, the tumors were heavily populated with non-proliferating polyploid/multinucleated giant cells that accumulated BrdUrd in their nuclei; the mean nucleus surface area of cells in these tumors (~397 mm^2^) was approximately 10 times higher than that in the tumor cells of untreated control rats (~43 mm^2^).
- Tumor regrowth began at ~35 days after cisplatin treatment and was driven by a small fraction of small, diploid, rapidly proliferating cells that had derived from polyploid/multinucleated giant cells.
- When these small cells (derived from giant cells) were injected into rats, the resulting tumors were refractory to cisplatin therapy.

The work of Puig et al. was published in 2008 [7]. Since then, several groups have confirmed that polyploid/multinucleated giant cancer cells can initiate tumors in vivo via depolyploidization/nuclear budding and that are resistant to cytotoxic therapy (e.g., [31,32,42,43]). Some of these studies involved cancer specimens from patients with different malignancies (reviewed in [12,32]). Zhang et al. [32], for example, examined the existence of polyploid/multinucleated giant cells in benign and malignant ovarian cancer. Giant cells were observed in serous cystadenoma, high-grade serous ovarian carcinoma, and metastatic high-grade serous ovarian carcinoma. The number of giant cells increased with an increasing disease stage and tumor grade. Furthermore, giant cells that were purified from patient tumors were capable of initiating tumors in vivo and exhibited resistance to cisplatin therapy. 

## 5. Giant Cancer Cells and Their Descendants Are Not Accounted for in Conventional Preclinical Assays

The progeny of polyploid/multinucleated giant cells might not arise until several weeks after genotoxic insult. Specifically, polyploid/multinucleated giant cells are fully manifested within ~3 days after anticancer treatment [12,13,14]. Although their depolyploidization/nuclear budding processes can commence at any time thereafter, it can take several weeks (if not months) until a stable, rapidly proliferating population of daughter cells emerges [7,32,50]. Accordingly, such cells cannot be accounted for in widely used cell-based assays, including the “long-term” (two week) colony formation assay, which is considered as the gold standard for radiosensitivity/chemosensitivity assessment. 

According to the work of Puig et al. [7] (also see Figure 7), polyploid/multinucleated giant cells and their tumor repopulating descendants might not be accounted for in conventional in vivo tumor growth delay assays unless the experiments are extended to beyond ~35 days after anticancer treatment. However, most anticancer drug discovery approaches involving live animals do not extend beyond this critical time point post-treatment.

## 6. Conclusions

Studies with animal models and clinical samples have demonstrated that most solid tumors contain a small proportion of giant cells with a highly enlarged nucleus or multiple nuclei. Although giant cells often cease to proliferate, they are associated with metastasis and exhibit resistance to anticancer therapy. The proportion of giant cells can increase markedly in response to genotoxic stress. Such cells enter a state of dormancy (proliferation arrest), during which they undergo depolyploidization and/or nuclear budding and give rise to stem cell-like progeny that repopulate the tumor. Giant cells can also promote stemness of surrounding cells through cell-cell tunneling.

Giant cells are often scored as “dead” in the widely used preclinical radiosensitivity/chemosensitivity assays (e.g., in vitro colony formation; in vivo tumor growth delay), because they cease to proliferate; similarly, the progeny of giant cells might not be accounted for in such assays simply because they emerge several weeks (if not months) post-treatment.

It is becoming increasingly evident that targeting giant cells before they can promote tumor repopulation might be an effective strategy in the war against cancer. To this end, Coward and Harding [21] discussed the possibility of targeting giant cells using pharmacological modulators of metabolic pathways. In addition, we have demonstrated that the treatment of giant cancer cells with pharmacological activators of apoptosis (e.g., sodium salicylate) triggers their demise [12]. There is also evidence that giant cells might be highly sensitive to the traditional Chinese medicine herbal mixture LQ. The latter conclusion is based on the work of Zhang et al. [42], which is discussed above (Section 3), demonstrating that the PC-3-GFP-LY cell line, which is highly enriched in giant cells, is resistant to conventional chemotherapeutic drugs but surprisingly exhibits a sensitivity to LQ that is comparable to the parental PC-3 cells. These proof-of-principle observations are encouraging and warrant further preclinical evaluation in animal models with experimental designs that account for the generation of polyploid/multinucleated giant cancer cells and their tumor repopulating descendants.

## References

[1] Enoch T., Norbury C. (1995). Cellular responses to DNA damage: Cell-cycle checkpoints, apoptosis and the roles of p53 and ATM. Trends Biochem. Sci..

[2] Meyn M.S. (1995). Ataxia-telangiectasia and cellular responses to DNA damage. Cancer Res..

[3] Ko L.J., Prives C. (1996). p53: Puzzle and paradigm. Genes Dev..

[4] Mirzayans R., Andrais B., Kumar P., Murray D. (2016). The growing complexity of cancer cell response to DNA-damaging agents: Caspase 3 mediates cell death or survival?. Int. J. Mol. Sci..

[5] Mirzayans R., Andrais B., Kumar P., Murray D. (2017). Significance of wild-type p53 signaling in suppressing apoptosis in response to chemical genotoxic agents: Impact on chemotherapy outcome. Int. J. Mol. Sci..

[6] Puck T.T., Marcus P.I. (1956). Action of X-rays on mammalian cells. J. Exp. Med..

[7] Puig P.E., Guilly M.N., Bouchot A., Droin N., Cathelin D., Bouyer F., Favier L., Ghiringhelli F., Kroemer G., Solary E. (2008). Tumor cells can escape DNA-damaging cisplatin through DNA endoreduplication and reversible polyploidy. Cell Biol. Int..

[8] Crescenzi E., Palumbo G., de Boer J., Brady H.J. (2008). Ataxia telangiectasia mutated and p21CIP1 modulate cell survival of drug-induced senescent tumor cells: Implications for chemotherapy. Clin. Cancer Res..

[9] Sliwinska M.A., Mosieniak G., Wolanin K., Babik A., Piwocka K., Magalska A., Szczepanowska J., Fronk J., Sikora E. (2009). Induction of senescence with doxorubicin leads to increased genomic instability of HCT116 cells. Mech. Ageing Dev..

[10] Mosieniak G., Sliwinska M.A., Alster O., Strzeszewska A., Sunderland P., Piechota M., Was H., Sikora E. (2015). Polyploidy formation in doxorubicin-treated cancer cells can favor escape from senescence. Neoplasia.

[11] Was H., Czarnecka J., Kominek A., Barszcz K., Bernas T., Piwocka K., Kaminska B. (2018). Some chemotherapeutics-treated colon cancer cells display a specific phenotype being a combination of stem-like and senescent cell features. Cancer Biol. Ther..

[12] Mirzayans R., Andrais B., Scott A., Wang Y.W., Kumar P., Murray D. (2017). Multinucleated giant cancer cells produced in response to ionizing radiation retain viability and replicate their genome. Int. J. Mol. Sci..

[13] Mirzayans R., Andrais B., Murray D. (2017). Impact of premature senescence on radiosensitivity measured by high throughput cell-based assays. Int. J. Mol. Sci..

[14] Mirzayans R., Andrais B., Murray D. (2017). Do multiwell plate high throughput assays measure loss of cell viability following exposure to genotoxic agents?. Int. J. Mol. Sci..

[15] Murray D., Mirzayans R. (2013). Role of therapy-induced cellular senescence in tumor cells and its modification in radiotherapy; the good, the bad and the ugly. J. Nucl. Med. Radiat. Ther..

[16] Kaur E., Rajendra J., Jadhav S., Shridhar E., Goda J.S., Moiyadi A., Dutt S. (2015). Radiation-induced homotypic cell fusions of innately resistant glioblastoma cells mediate their sustained survival and recurrence. Carcinogenesis.

[17] Roninson I.B. (2003). Tumor cell senescence in cancer treatment. Cancer Res..

[18] Sikora E., Mosieniak G., Sliwinska M.A. (2016). Morphological and functional characteristic of senescent cancer cells. Curr. Drug Targets.

[19] Galmarini C.M., Falette N., Tabone E., Levrat C., Britten R., Voorzanger-Rousselot N., Roesch-Gateau O., Vanier-Viornery A., Puisieux A., Dumontet C. (2001). Inactivation of wild-type p53 by a dominant negative mutant renders MCF-7 cells resistant to tubulin-binding agent cytotoxicity. Br. J. Cancer.

[20] Li Q., Rycaja K., Chena X., Tanga D.G. (2015). Cancer stem cells and cell size: A causal link?. Semin. Cancer Biol..

[21] Coward J., Harding A. (2014). Size does matter: Why polyploid tumor cells are critical drug targets in the war on cancer. Front. Oncol..

[22] Solari F., Domenget C., Gire V., Woods C., Lazarides E., Rousset B., Jurdic P. (1995). Multinucleated cells can continuously generate mononucleated cells in the absence of mitosis: A study of cells of the avian osteoclast lineage. J. Cell Sci..

[23] Walen K.H. (2002). The origin of transformed cells: Studies of spontaneous and induced cell transformation in cell cultures from marsupials, a snail, and human amniocytes. Cancer Genet..

[24] Sundaram M., Guernsey D.L., Rajaraman M.M., Rajaraman R. (2004). Neosis: A novel type of cell division in cancer. Cancer Biol. Ther..

[25] Walen K.H. (2004). Spontaneous cell transformation: Karyoplasts derived from multinucleated cells produce new cell growth in senescent human epithelial cell cultures. Cell. Dev. Biol. Anim..

[26] Rajaraman R., Rajaraman M.M., Rajaraman S.R., Guernsey R.L. (2005). Neosis—A paradigm of self-renewal in cancer. Cell Biol. Int..

[27] Walen K.H. (2005). Budded karyoplasts from multinucleated fibroblast cells contain centrosomes and change their morphology to mitotic cells. Cell Biol. Int..

[28] Rajaraman R., Guernsey D.L., Rajaraman M.M., Rajaraman S.R. (2006). Stem cells, senescence, neosis and self-renewal in cancer. Cell Biol. Int..

[29] Jiang Q., Zhang Q., Wang S., Xie S., Fang W., Liu Z., Liu J., Yao K. (2015). A fraction of CD133+ CNE2 cells is made of giant cancer cells with morphological evidence of asymmetric mitosis. J. Cancer.

[30] Esmatabadi M.J., Bakhshinejad B., Motlagh F.M., Babashah S., Sadeghizadeh M. (2016). Therapeutic resistance and cancer recurrence mechanisms: Unfolding the story of tumour coming back. J. Biosci..

[31] Lv H., Shi Y., Zhang L., Zhang D., Liu G., Yang Z., Li Y., Fei F., Zhang S. (2014). Polyploid giant cancer cells with budding and the expression of cyclin E, S-phase kinase-associated protein 2, stathmin associated with the grading and metastasis in serous ovarian tumor. BMC Cancer.

[32] Zhang S., Mercado-Uribe I., Xing Z., Sun B., Kuang J., Liu J. (2014). Generation of cancer stem-like cells through the formation of polyploid giant cancer cells. Oncogene.

[33] Fei F., Zhang D., Yang Z., Wang S., Wang X., Wu Z., Wu Q., Zhang S. (2015). The number of polyploid giant cancer cells and epithelial-mesenchymal transition-related proteins are associated with invasion and metastasis in human breast cancer. J. Exp. Clin. Cancer Res..

[34] Zhang S., Zhang D., Yang Z., Zhang X. (2016). Tumor budding, micropapillary pattern, and polyploidy giant cancer cells in colorectal cancer: Current status and future prospects. Stem Cells Int..

[35] Mittal K., Donthamsetty S., Kaur R., Yang C., Gupta M.V., Reid M.D., Choi D.H., Rida P.C.G., Aneja R. (2017). Multinucleated polyploidy drives resistance to Docetaxel chemotherapy in prostate Cancer. Br. J. Cancer.

[36] Niu N., Zhang J., Zhang N., Mercado-Uribe I., Tao F., Han Z., Pathak S., Multani A.S., Kuang J., Yao J. (2016). Linking genomic reorganization to tumor initiation via the giant cell cycle. Oncogenesis.

[37] Erenpreisa J.A., Cragg M.S., Fringes B., Sharakhov I., Illidge T.M. (2000). Release of mitotic descendants by giant cells from irradiated Burkitt’s lymphoma cell lines. Cell Biol. Int..

[38] Illidge T.M., Cragg M.S., Fringes B., Olive P., Erenpresia J.A. (2000). Polyploid giant cells provide a survival mechanism of p53 mutant cells after DNA damage. Cell Biol. Int..

[39] Erenpreisa J., Cragg M.S. (2013). Three steps to the immortality of cancer cells: Senescence, polyploidy and self-renewal. Cancer Cell Int..

[40] Erenpreisa J., Salmina K., Huna A., Jackson T.R., Vazquez-Martin A., Cragg M.S. (2015). The “virgin birth”, polyploidy, and the origin of cancer. Oncoscience.

[41] Díaz-Carballo D., Saka S., Klein J., Rennkamp T., Acikelli A.H., Malak S., Jastrow H., Wennemuth G., Tempfer C., Schmitz I. (2018). A distinct oncogenerative multinucleated cancer cell serves as a source of stemness and tumor heterogeneity. Cancer Res..

[42] Zhang L., Wu C., Hoffman R.M. (2015). Prostate cancer heterogeneous high-metastatic multi-organ-colonizing chemo-resistant variants selected by serial metastatic passage in nude mice are highly enriched for multinucleate giant cells. PLoS ONE.

[43] Weihua Z., Lin Q., Ramoth A.J., Fan D., Fidler I.J. (2011). Formation of solid tumors by a single multinucleated cancer cell. Cancer.

[44] Hasegawa K., Suetsugu A., Nakamura M., Matsumoto T., Aoki H., Kunisada T., Shimizu M., Saji S., Moriwaki H., Hoffman R.M. (2017). Imaging the role of multinucleate pancreatic cancer cells and cancer-associated fibroblasts in peritoneal metastasis in mouse models. Anticancer Res..

[45] Zhang D., Yang X., Yang Z., Fei F., Li S., Qu J., Zhang M., Li Y., Zhang X., Zhang S. (2017). Daughter cells and erythroid cells budding from PGCCs and their clinicopathological significances in colorectal cancer. J. Cancer.

[46] Niu N., Mercado-Uribe I., Liu J. (2017). Dedifferentiation into blastomere-like cancer stem cells via formation of polyploid giant cancer cells. Oncogene.

[47] Shu Z., Row S., Deng W.M. (2018). Endoreplication: The good, the bad, and the ugly. Trends Cell Biol..

[48] Zheng L., Dai1 H., Zhou M., Li X., Liu C., Guo Z., Wu X., Wu J., Wang C., Zhong J. (2012). Polyploid cells rewire DNA damage response networks to overcome replication stress-induced barriers for tumour progression. Nat. Commun..

[49] Ogdena A., Ridaa P.C.G., Knudsenb B., Kucukc O., Anejaa R. (2015). Docetaxel-induced polyploidization may underlie chemoresistance and disease relapse. Cancer Lett..

[50] Vitale I., Senovilla L., Jemaà M., Michaud M., Galluzzi L., Kepp O., Nanty L., Criollo A., Rello-Varona S., Manic G. (2010). Multipolar mitosis of tetraploid cells: Inhibition by p53 and dependency on Mos. EMBO J..

